# Viral Loads in Clinical Specimens and SARS Manifestations

**DOI:** 10.3201/eid1009.040058

**Published:** 2004-09

**Authors:** I.F.N. Hung, V.C.C. Cheng, A.K.L. Wu, B.S.F. Tang, K.H. Chan, C.M. Chu, M.M.L. Wong, W.T. Hui, L.L.M. Poon, D.M.W. Tse, K.S. Chan, P.C.Y. Woo, S.K.P. Lau, J.S.M. Peiris, K.Y. Yuen

**Affiliations:** * Queen Mary Hospital at the University of Hong Kong, Hong Kong Special Administrative Region (SAR), People's Republic of China;; † Prince of Wales Hospital, Hong Kong SAR, People's Republic of China;; ‡ United Christian Hospital, Hong Kong SAR, People's Republic of China;; § Caritas Medical Centre, Hong Kong SAR, People's Republic of China

**Keywords:** SARS, viral load, RT-qPCR, research

## Abstract

A retrospective viral load study was performed on clinical specimens from 154 patients with laboratory-confirmed severe acute respiratory syndrome (SARS); the specimens were prospectively collected during patients' illness. Viral load in nasopharyngeal aspirates (n = 142) from day 10 to day 15 after onset of symptoms was associated with oxygen desaturation, mechanical ventilation, diarrhea, hepatic dysfunction, and death. Serum viral load (n = 53) was associated with oxygen desaturation, mechanical ventilation, and death. Stool viral load (n = 94) was associated with diarrhea, and urine viral load (n = 111) was associated with abnormal urinalysis results. Viral replications at different sites are important in the pathogenesis of clinical and laboratory abnormalities of SARS.

## Abstract 2

The number of anatomical sites with detectable viral loads by RT-qPCR appeared to correlate with death risk.

Severe acute respiratory syndrome (SARS) is an emerging infectious disease that affected 8,098 persons and caused 774 deaths from November 1, 2002, to September 26, 2003 (*1*). A novel coronavirus (SARS-CoV) was consistently isolated from SARS patients in three different continents independently (*2**–**4*). Animal models using macaque monkeys, ferrets, and domestic cats were established (*5**–**7*); no extrapulmonary lesions could be identified in these animals, although virus isolation and reverse transcription–polymerase chain reaction (RT-PCR) results for viral RNA from pharyngeal secretions, tracheobronchial secretions, urine, rectal swabs or stool, kidney tissue or lung tissues were positive.

Recently, we reported the use of RT-PCR to detect SARS-CoV RNA from nasopharyngeal aspirates (NPA) and throat swab, urine, and stool specimens (*8*). We also developed quantitative RT-PCR (RT-qPCR) assays using the LightCycler System (Idaho Technology Inc., Idaho Falls, ID) to augment the sensitivity of detection (*9*). The serial viral load in NPA was also used to monitor clinical progress and response to antiviral therapy, and viral load in serum on admission was used as a marker of prognosis (*10**–**12*). Extrapulmonary manifestations, such as hematologic changes, diarrhea, and liver dysfunction are common in SARS patients but not in animal models (*13**–**15*). In this study, we assayed and analyzed viral load in clinical specimens taken from different anatomical sites from day 10 to 15 after the onset of symptoms to understand the role of SARS-CoV in the pathogenesis of clinical manifestations and laboratory test abnormalities in SARS patients.

## Patients and Methods

We included in this quantitative virologic study 154 patients who fulfilled the modified World Health Organization (WHO) definition of SARS and whose treatment was managed in the United Christian Hospital and Caritas Medical Centre of Hong Kong Special Administration Region of China. All patients' infections were either serologically confirmed (fourfold rise of indirect immunofluorescent antibody titer in serum taken on admission and within 28 days of symptom onset) or RT-PCR was positive for SARS-CoV RNA (confirmed from their clinical specimens for those who died or did not seroconvert before day 28). The case definition includes temperature of >38°C, cough or shortness of breath, and new pulmonary infiltrates shown on chest x-ray or high-resolution computed tomographic scan in the absence of an alternative diagnosis to explain the clinical signs and symptoms.

The treatment protocol, clinical manifestations, and progression of disease in part of this cohort had been previously reported (*10**,**11**,**16*). In brief, patients were prospectively monitored for diarrhea, oxygen desaturation, mechanical ventilation; laboratory evidence of lymphopenia, renal impairment, liver dysfunction, or abnormal urinalysis during the first 15 days; and death. Diarrhea was defined as bowel movements >3 times per day for 2 consecutive days. Oxygen desaturation was defined as <90% oxygen saturation measured by pulse oximetry while breathing room air. Some of these patients later required mechanical ventilation. Hepatic dysfunction was defined as a mean level of alanine aminotransferase (ALT), alkaline phosphatase (ALP), or both, greater than the upper limit of normal from day 10 to day 15 after onset of symptoms. Impaired renal function was defined as a serum creatinine level higher than the reference range on 2 consecutive days. Lymphopenia was defined as an absolute lymphocyte count <1,000/ µL on 2 consecutive days. Abnormal urinalysis results were defined as proteinuria (>30 mg/dL), microscopic hematuria (>10 erythrocytes/µL), pyuria (>50 leukocytes/µL) on a dipstick test, or casts in urine (Combur Test, Roche Diagnostics GmbH, Mannheim, Germany) examined with inverted microscope by an experienced technician.

To diagnose SARS-CoV infection, NPA and serum samples were taken on admission. Convalescent-phase serum samples were taken between days 7 and 28 after symptom onset. In all patients, RT-PCR for SARS-CoV RNA was performed on the NPA collected on admission. RT-qPCR was performed for patients who had NPA, serum, stool, and urine specimens collected on days 10 to 15 after the onset of symptoms. All virologic diagnostic laboratory tests, including viral culture, RT-PCR, RT-qPCR, and immunofluorescent antibody detection for immunoglobulin (Ig) G, were performed according to our previously published protocols (*2**,**10*). NPA was obtained by suction through both nostrils with a Pennine 6 mucus extractor (Pennine Healthcare, Derby, UK) and mucus specimen trap (MST-7000, Pennine Healthcare). The catheter was connected midway between the tip of the nose and the auditory meatus; it was rotated continuously and slowly retrieved with intermittent suction at a negative pressure of 100 mm Hg for 15 s. The procedure was repeated in the other nostril. Secretions stuck to the lumen of the catheter were transferred to the mucus trap by flushing with 1 mL of viral transport medium, which consists of Earle's Balanced Salt Solution (BioSource International, Camarillo, CA), 4.4% bicarbonate, 5% bovine serum albumin, vancomycin (100 µg/mL), amikacin (30 µg/mL), and nystatin (40 U/mL). A 10% stool suspension was made by swirling approximately 1 g of stool in 10 mL of viral transport medium. Midstream urine was collected in a sterile container. Serum from clotted blood was collected and stored at 4°C for antibody testing and at –20°C before RNA extraction. The other specimens were stored at 4°C before RNA extraction.

RNA from clinical samples was extracted by using the QIAamp virus RNA mini kit (Qiagen, Cologne, Germany) as instructed by the manufacturer. For all specimens, 140 µL of the sample was used for RNA extraction, and extracted RNA was finally eluted in 30 µL of RNase-free water and stored at –20°C. For the RT-qPCR assay, RNA and cDNA were generated as described (*9*). cDNA was amplified in a 7000 Sequence Detection System (Applied Biosystems, Foster City, CA) by using TaqMan PCR Core Reagent kit (Applied Biosystems). In a typical reaction, 2 µL of cDNA was amplified in a 25-µL reaction containing 0.625 U AmpliTaq Gold polymerase (Applied Biosystems), 2.5 µL of 10x TaqMan buffer A, 0.2 mmol/L of dNTPs, 5.5 mmol/L of MgCl_2_, 2.5 U of AmpErase UNG, and 1x primers-probe mixture (Assays by Design, Applied Biosystems). The forward primer was 5´-CAGAACGCTGTAGCTTCAAAAATCT-3´ corresponding to nt 17,718–17,742 of the SARS-CoV genome), and the reverse primer was 5´-TCAGAACCCTGTGATGAATCAACAG-3´ (corresponding to nt 17,761–17,785). The sequence of the reporter probe was 5´-(FAM)TCTGCGTAGGCAATCC(NFQ)-3´ (FAM, 6-carboxyfluorescein; NFQ, nonfluorescent quencher; complementary to nt 17,745–17,760). Reactions were first incubated at 50°C for 2 min, followed by 95°C for 10 min. Reactions were then thermocycled for 40 cycles (95°C for 15 s, 60°C for 1 min). Plasmids containing the target sequences were used as standard controls. To monitor the integrity of RNA extraction for each sample, a housekeeping gene, β-actin, was detected by real-time RT-PCR with two primers: β-actin forward, 5´-CCCAAGGCCAACCGCGAGAAGAT-3´ and reverse, 5´-GTCCCGGCCAGCCAGGTCCAG-3´. All samples were found to contain detectable β-actin RNA.

All timed data were calculated from onset of symptoms. We compared the viral load in these specimens with the presence or absence of diarrhea, oxygen desaturation, mechanical ventilation, lymphopenia, hepatic dysfunction, abnormal urinalysis findings, and death rate by chi-square or Fisher exact test for categorical variables and by Mann-Whitney U test for continuous variables. A two-tailed p value < 0.05 was considered significant. Correlation between the number of anatomic sites with detectable viral load by RT-qPCR and death rate was calculated by linear regression. SPSS (version 11.0, SPSS Inc., Chicago, IL) was used for all analyses.

## Results

Of the 154 SARS patients who satisfied the WHO definition for SARS, their age range was 20–80 years (mean 41.5 years). The male-to-female ratio was 0.60, and 31 (20.1%) were healthcare workers. One hundred and fifty patients (97.4%) were ethnic Chinese, and 4 (2.6%) were Filipino. The following clinical specimens taken from days 10 to 15 from individual patients were available: NPA (n = 142), serum (n =53), stool (n = 94), and urine (n = 111). All patients had laboratory-confirmed SARS, either virologically by qualitative RT-PCR in 116 (75.3%) patients (from 30.5% of NPA, 68.2% of stool samples, and 26.6% of urine samples), by RT-qPCR in 126 (81.8%) patients (from 42.3% of NPA, 41.5% of serum samples, 87.2% of stool samples, and 28.8% of urine samples), or by seroconversion in indirect immunofluorescent antibody assay in 136 (88.3%) patients. Six patients who died before day 28 did not have serologic documentation, and 12 others had not seroconverted by day 28, but all had SARS confirmed by RT-PCR. Viral cultures were positive for SARS-CoV in 20 patients from NPA (n = 18), stool (n = 1), and urine (n = 1).

Besides fever and pulmonary infiltrates shown on x-ray, peripheral blood lymphopenia was the most common hematologic abnormality on admission and afterwards (Table 1). From admission to day 10, the peripheral lymphocyte count in all patients decreased. From days 10 to 19, the peripheral lymphocyte count continued to decrease in patients who ultimately died, whereas in those who survived, lymphocyte count gradually increased (Figure). Seroconversion to SARS-CoV antibodies also started at around day 10. Diarrhea (43.5%) was the most common extrapulmonary clinical manifestation from days 10 to 15, followed by hepatic dysfunction (39.0%). Abnormal urinalysis was found in 87 (56.4%) patients, with increased leukocyte esterase in 49.4%, pyuria in 23%, microscopic hematuria in 20.1%, and proteinuria in 3.8%. None of the patients had impaired renal function from admission to day 20. Oxygen desaturation developed in 73 patients (47.4%) at a mean of 10.2 (standard deviation [SD] = 5.1) days, and 34 patients (22.1%) died from SARS a mean of 32.4 (SD = 10.6) days after onset of symptoms. Only four patients were found to have abnormal mean ALP levels from admission to day 20. Therefore, only the mean ALT level from days 10 to 20 was analyzed for correlation with viral load. The mean ALT level between admission and day 10 was also analyzed. However, no significant association was found between the mean ALT level and the viral loads from different specimens.

**Table 1:** The dominant clinical features and laboratory abnormalities of 154 patients with SARS^a^

Clinical feature	On admission (%)	Days 10–15 (%)
Oxygen desaturation	2 (1.4)	73 (47.4)
Diarrhea	15 (10.6)	60 (39.0)
Lymphopenia	105 (68.1)	126 (81.8)
Hepatic dysfunction	34 (22.1)	87 (56.4)
Abnormal urinalysis findings	NA	67 (43.5)

^a^SARS, severe acute respiratory syndrome; NA, not applicable.

**Figure:**
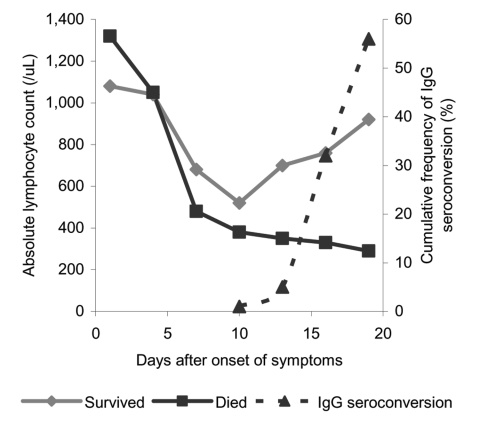
Serial change of the mean absolute lymphocyte count and immunoglobulin (Ig) G seroconversion of severe acute respiratory syndrome (SARS)–associated coronavirus in 154 SARS patients.

Both the positivity rate (87.2%) and the viral load by RT-qPCR (6.1 log_10_ copies/mL) were highest for stool specimens, followed by NPA (42.3% and 2.4 log_10_ copies/mL, respectively). Although virus was more frequently detected in serum (41.5%) than urine (28.8%), their mean viral loads for those specimens were 2.7 log_10_ copies/mL and 4.4 log_10_ copies/mL, respectively (Table 2). More patients with lymphopenia on admission had virus detectable by RT-qPCR in NPA (p = 0.04, odds ratio [OR] 2.2, 95% confidence interval [CI] 1.7–4.4, data not shown). Both the positivity rate and the viral load in NPA were significantly correlated with diarrhea, oxygen desaturation, mechanical ventilation, hepatic dysfunction, and death rate (all p < 0.01) (Table 3 and Table 4). A high viral load in serum was also associated with oxygen desaturation, mechanical ventilation, hepatic dysfunction, and death (all p < 0.01) but not with diarrhea or abnormal urinalysis findings. Stool viral load was associated with diarrhea, hepatic dysfunction, abnormal urinalysis results, and death (all p < 0.01). A higher viral load in urine was significantly correlated with abnormal urinalysis and with diarrhea (both p < 0.01). These findings were confirmed by subgroup analysis of 40 patients with specimen samples taken from all four sites (NPA, serum, stool, and urine) (Table 5 and Table 6). Again, a detectable viral load in NPA and serum was associated with oxygen desaturation (OR 5.3, 95% CI 1.4–21.1), mechanical ventilation (OR 1.6, 95% CI 1.1–2.4), and death (OR 1.8, 95% CI 1.2–2.7). The stool viral load was associated with diarrhea (OR 18, 95% CI 2.0–16.3), although a higher viral load in urine was no longer associated with abnormal urinalysis results.

**Table 2:** SARS-CoV and viral load in different clinical specimens taken during days 10 to 15 after the onset of symptoms^a^

Clinical specimens	Mean viral load in log_10_ copies/mL (SD), all specimens	Mean viral load in log_10_ copies/mL (SD), positive specimens only	Positivity by RT-qPCR (%)
NPA (n = 142)	2.4 (3.1)	5.8 (1.7)	60/142 (42.3)
Serum (n = 53)	1.1 (1.4)	2.7 (0.6)	22/53 (41.5)
Stool (n = 94)	6.1 (3.0)	7.0 (2.1)	82/94 (87.2)
Urine (n = 111)	1.3 (2.1)	4.4 (1.3)	32/111 (28.8)

^a^SARS-CoV, severe acute respiratory syndrome–associated coronavirus; SD, standard deviation; RT-qPCR, quantitative reverse transcription–polymerase chain reaction; NPA, nasopharyngeal aspirates.

**Table 3:** Correlation of RT-qPCR of clinical specimens taken during days 10 to 15 and clinical manifestations in 154 patients with SARS^a^

Specimen	Diarrhea			O_2_ desaturation			Mechanical ventilation			Death		
	Y	N	p value; OR (CI)	Y	N	p value; OR (CI)	Y	N	p value; OR (CI)	Y	N	p value; OR (CI)
NPA (n = 142)												
RT-qPCR (+) (n = 60)	37	23	< 0.01; 2.5 (1.3–5.0)	37	23	< 0.01; 3.1 (1.6–6.2)	22	38	< 0.01; 11.3 (3.6–35.1)	24	36	< 0.01; 54 (7.1–415.0)
RT-qPCR (–) (n = 82)	32	50		28	54		4	78		1	81	
Median viral load (log_10_ copies/mL)	3.2	0	0.02	4.5	0	< 0.01	6.4	0	< 0.01	6.5	0	< 0.01
Serum (n = 53)												
RT-qPCR (+) (n = 22)	12	10	0.4	13	9	< 0.01; 5 (1.5–16.4)	7	15	< 0.01; 1.5 (1.1–2.0)	8	14	< 0.01; 17.1 (2.0–151.0)
RT-qPCR (–) (n = 31)	13	18		7	24		0	31		0	31	
Median viral load (log_10_ copies/mL)	0	0	0.5	2.3	0	< 0.01	2.6	0	< 0.01	2.6	0	< 0.01
Stool (n = 94)												
RT-qPCR (+) (n = 82)	46	36	< 0.01; 14.1 (1.7–114.0)	39	43	0.5	16	66	1	17	65	0.5
RT-qPCR (–) (n = 12)	1	11		4	8		2	10		1	11	
Median viral load (log_10_ copies/mL)	7.5	5	< 0.01	7.2	5	0.1	8	7	0.05	8.3	6.9	< 0.01
Urine (n = 111)												
RT-qPCR (+) (n = 32)	23	9	< 0.01; 3.8 (1.5–9.2)	15	17	0.8	7	25	0.4	7	25	0.4
RT-qPCR (–) (n = 79)	32	47		34	45		12	67		11	68	
Median viral load (log_10_ copies/mL)	0	0	< 0.01	0	0	0.7	0	0	0.3	0	0	0.4

^a^RT-qPCR, quantitative reverse transcription–polymerase chain reaction; SARS, severe acute respiratory syndrome; Y, yes; N, no; OR, odds ratio; CI, 95% confidence interval; NPA, nasopharyngeal aspirates.

**Table 4:** Correlation of RT-qPCR clinical specimens taken during days 10 to 15 and laboratory values in 154 patients with SARS^a^

Specimen	Lymphopenia			Hepatic dysfunction			Abnormal urinalysis results		
	Y	N	p value; OR (CI)	Y	N	p value; OR (CI)	Y	N	p value; OR (CI)
NPA (n = 142)									
RT-qPCR (+) (n = 60)	56	4	0.8	40	20	< 0.01; 2.5 (1.2-5.2)	43	17	0.07
RT-qPCR (–) (n = 82)	70	12		38	44		52	30	
Median viral load (log_10_ copies/mL)	0	0	0.3	2.7	0	< 0.01	0	0	0.1
Serum (n = 53)									
RT-qPCR (+) (n = 22)	20	2	0.7	12	10	1	18	4	1
RT-qPCR (–) (n = 31)	26	5		19	12		26	5	
Median viral load (log_10_ copies/mL)	0	0	0.4	0	0	0.9	0	0	0.5
Stool (n = 94)									
RT-qPCR (+) (n = 82)	75	7	0.2	46	36	0.1	62	20	0.2
RT-qPCR (–) (n = 12)	9	3		4	8		7	5	
Median viral load (log_10_ copies/mL)	7.2	8.2	0.5	7.7	6.6	< 0.01	7.6	3.1	< 0.01
Urine (n = 111)									
RT-qPCR (+) (n = 32)	29	3	0.7	20	12	0.2	29	3	< 0.01; 7.2 (1.6–32.9)
RT-qPCR (–) (n = 79)	69	10		38	41		50	29	
Median viral load (log_10_ copies/mL)	0	0	0.3	0	0	0.2	0	0	< 0.01

^a^RT-qPCR, quantitative reverse transcription–polymerase chain reaction; SARS, severe acute respiratory syndrome; Y, yes; N, no; OR, odds ratio; CI, 95% confidence interval; NPA, nasopharyngeal aspirates.

**Table 5:** Correlation of RT-qPCR clinical specimens taken during days 10 to 15 and clinical manifestations in 40 patients with SARS who had specimens taken from all four anatomic sites^a^

Specimen	Diarrhea			O_2_ desaturation			Mechanical ventilation			Death		
	Y	N	p value; OR (CI)	Y	N	p value; OR (CI)	Y	N	p value; OR (CI)	Y	N	p value; OR (CI)
NPA (n = 40)												
RT-qPCR (+) (n = 18)	11	7	0.4	11	7	0.02; 5.3 (1.4–21.1)	7	11	< 0.01; 1.6 (1.1–2.4)	8	10	< 0.01; 1.8 (1.2–2.7)
RT-qPCR (–) (n = 22)	10	12		5	17		0	22		0	22	
Median viral load (log_10_ copies/mL)	2.9	0	0.4	5.1	0	< 0.01	6.4	0	< 0.01	6.4	0	< 0.01
Serum (n = 40)												
RT-qPCR (+) (n = 18)	12	6	0.1	11	7	0.02; 5.3 (1.4–21.1)	7	11	< 0.01; 1.6 (1.1–2.4)	8	10	< 0.01; 1.8 (1.2–2.7)
RT-qPCR (–) (n = 22)	9	13		5	17		0	22		0	22	
Median viral load (log_10_ copies/mL)	2.3	0	0.3	2.4	0	0.02	2.6	0	< 0.01	2.6	0	< 0.01
Stool (n = 40)												
RT-qPCR (+) (n = 30)	20	10	< 0.01; 18 (2.0–16.3)	12	18	1	5	25	1	7	23	0.7
RT-qPCR (–) (n = 10)	1	9		4	6		2	8		1	9	
Median viral load (log_10_ copies/mL)	7.8	2.6	< 0.01	7.3	7.2	0.5	8.2	7.2	0.5	8.7	7	0.03
Urine (n = 40)												
RT-qPCR (+) (n = 9)	6	3	0.5	3	6	0.7	1	8	1	2	7	1
RT-qPCR (–) (n = 31)	15	16		13	18		6	25		6	25	
Median viral load (log_10_ copies/mL)	0	0	0.4	0	0	0.6	0	0	0.5	0	0	1

^a^RT-qPCR, quantitative reverse transcription–polymerase chain reaction; SARS, severe acute respiratory syndrome; Y, yes; N, no; OR, odds ratio; CI, 95% confidence interval; NPA, nasopharyngeal aspirates.

**Table 6:** Correlation of RT-qPCR clinical specimens taken during days 10 to 15 and laboratory values in 40 patients with SARS who had specimens taken from all four anatomic sites^a^

Specimen	Lymphopenia			Hepatic dysfunction			Abnormal urinalysis results		
	Y	N	p value	Y	N	p value	Y	N	p value
NPA (n = 40)									
RT-qPCR (+) (n = 18)	15	3	0.6	10	8	0.8	16	2	0.4
RT-qPCR (–) (n = 22)	20	2		11	11		16	6	
Median viral load (log_10_ copies/mL)	2.9	0	0.9	0	0	0.9	0.8	0	0.5
Serum (n = 40)									
RT-qPCR (+) (n = 18)	16	2	1	10	8	0.8	16	2	0.4
RT-qPCR (–) (n = 22)	19	3		11	11		16	6	
Median viral load (log_10_ copies/mL)	0	0	0.6	0	0	0.7	1.1	0	0.3
Stool (n = 40)									
RT-qPCR (+) (n = 30)	27	3	0.6	18	12	0.3	25	5	0.2
RT-qPCR (–) (n = 10)	8	2		3	7		6	4	
Median viral load (log_10_ copies/mL)	7.9	7.2	0.8	7.8	4.7	0.04	7.8	2.6	0.1
Urine (n = 40)									
RT-qPCR (+) (n = 9)	8	1	1	7	2	0.1	8	1	0.7
RT-qPCR (–) (n = 31)	27	4		14	17		23	8	
Median viral load (log_10_ copies/mL)	0	0	1	0	0	0.06	0	0	0.4

^a^RT-qPCR, quantitative reverse transcription–polymerase chain reaction; SARS, severe acute respiratory syndrome; Y, yes; N, no; NPA, nasopharyngeal aspirates.

In the subgroup analysis of 40 patients who had all types of specimen available for analysis, 34 had antibody seroconversion, and 6 died before day 28 without serologic documentation. Diagnosis of each was confirmed by qualitative RT-PCR. In this group, the number of anatomic sites with positive RT-qPCR strongly correlated with the risk for death (Pearson correlation = 0.517, p < 0.0005). No deaths were associated with negative RT-qPCR at all sites or just one positive site. Risk for death was 12.5% with positivity at two sites and 41.7% with positivity at three sites. The death rate increased to 66.7% if RT-qPCR was positive at all four sites.

## Discussion

Viral load reflects the dynamic interaction between viral replication and clearance by body defense mechanisms. Examining viral load in SARS patients has been used to diagnose and monitor progress or response to antiviral therapy (*10**,**11*). We have shown that viral load in NPA peaked around day 10 (*10*), with a rapid decrease and concomitant normalization of lymphocyte count and rise in serum antibodies to SARS-CoV (Figure). Presence of virus and viral load in different body fluids may also bear on possible modes of transmission. Infectivity at day 10, reflected by a mean peak viral load of 5.8 log _10_ copies/mL and 7.0 log _10_ copies/mL in positive specimens of NPA and stool, respectively, suggested that respiratory droplets and indirect contact with feces may be important mechanisms of transmission. Viral load has typically been measured in NPA and serum at admission as a diagnostic and prognostic tool (*10**,**12**,**17*). Viral load in body fluids other than NPA and serum has not been studied to elucidate transmission and pathogenesis of SARS.

The importance of SARS-CoV as a respiratory pathogen is supported by the strong association of viral load in NPA with oxygen desaturation, mechanical ventilation, and death. Unexpectedly, viral load in NPA was also associated with diarrhea and hepatic dysfunction. Anecdotal reports of the use of steroids to treat SARS tend to suggest these extrapulmonary manifestations could be part of an inflammatory "spillover" from virus-induced immune dysfunction or excessive cytokine activation in the lungs (*18*). However, our findings suggest that viral replication in extrapulmonary sites may be important, since viral load in stool is highly correlated with diarrhea, and electron microscopy of the ileum and colonic biopsy from SARS patients showed numerous intracellular and extracellular virus particles (*19*). High viral load in urine is also associated with abnormal urinalysis findings. In this regard, SARS-CoV is currently known to grow only in fetal monkey kidney cells (i.e., fRhK4 or Vero E6) and the colonic carcinoma cell line (CACO-2). The correlation of viral load in stool with hepatic dysfunction is not completely unexpected, since high viral load in the stool is likely to be associated with significant portal venous viremia. In fact, viral load in stool is also associated with death.

Serum viral load correlates with oxygen desaturation, mechanical ventilation, and death. This finding is not surprising, since viremia has also been reported in adenovirus, respiratory syncytial virus (RSV), and rotavirus infections (*20**–**22*). However, viremia is reported to be short-lasting in mucosal infections. In one study, 5 out of 41 neonates with positive RSV antigen in nasal wash specimens were positive for RSV RNA in blood (*21*). High levels of adenovirus DNA in serum was also associated with death in children in whom adenovirus infection developed after allogeneic stem-cell transplantation. Six (86%) of 7 children who died of adenovirus infection, compared with 2 (7%) of 29 other patients, had high serum levels of adenoviral DNA (p < 0.0001) (*20*). The lack of association between viral load and lymphopenia at day 10 can be explained by the routine use of steroids, which induce apoptosis of lymphocytes. The apparently inferior performance of serum viral load as a prognostic indicator could be related to fewer available serum samples in this cohort. However, 38% of this group of 53 patients had oxygen desaturation; this proportion is not significantly different from the 142 patients (46%) who had submitted nasopharyngeal samples from day 10 to day 15.

Compared with other common viral respiratory diseases, the onset of peak viral load in the nasopharynx of SARS patients appears to be delayed. In a prospective study of viral shedding in nasopharyngeal secretions in experimental adult RSV infections, as measured by 50% tissue culture infective dose (TCID_50_) viral titer or RT-qPCR, RSV is detected from day 2 to day 12, with a plateau phase from day 3 to day 8, at a peak viral load of 5 log_10_ copies/mL (*23*). In the case of experimental adult influenza, viral replication in NPA peaked ≈48 hours after the onset of symptoms and declined sharply thereafter, with an insignificant amount of viral shedding after day 8. Peak virus titers in symptomatic volunteers infected with influenza A H3N2 were 10^2.5^-10^7.0^ TCID_50_/mL of nasopharyngeal wash. Viral load was positively correlated with symptoms of fever and malaise as well as the amount of viral shedding (*24*). However, the reported low incidence of viremia and the early peak nasopharyngeal viral load in these two conditions could be explained by inherent characteristics of viral replication, background IgG and IgA against cross-reactive homologous antigens from previous infections, or innate immunity of the host. In many of these experimental infections in which the profile of viral load in NPA was documented, volunteers were adults who had low-level background antibodies and concomitant cell-mediated immunity against influenza or RSV (*23**,**24*). Falsely negative serum viral load in influenza or RSV infection could be related to hemagglutinating properties of these two viruses, which may remain stuck on the erythrocyte in the clotted blood sample. In a novel emerging infectious disease such as SARS, most of the general population would not have background or partial immunity (*2**,**25*); viremia or a delayed peak viral load at day 10 in NPA is therefore not completely unexpected.

One limitation of the present study is its retrospective nature. Only specimens provided at approximately day 10 could be tested and analyzed. Changes of lymphocyte subsets were also not analyzed. Nonetheless, lymphocyte changes in SARS patients were well reported by two other groups who showed a consistent decrease in the peripheral blood level of dendritic cell subsets, natural killer cells, CD4+ and CD8+ T lymphocytes, and B lymphocytes (*26**,**27*).

SARS is predominantly a respiratory infection, which possibly spreads through the mucosal lumen or the bloodstream to extrapulmonary sites where viral replication leads to nonrespiratory manifestations. Concomitant immune dysregulation and associated inflammatory damage could accentuate disease progression and death. High viral load in NPA, with or without high viral load in serum, is a useful prognostic indicator of respiratory failure or death. The presence of viral RNA in multiple body sites also indicates poor prognosis. Early treatment with an effective antiviral agent before day 10 may decrease the peak viral load, ameliorate symptoms, and improve outcome; early treatment may also reduce viral shedding and thus the risk for transmission.
